# Dendritic cells change IL-27 production pattern during childhood

**DOI:** 10.1186/s13104-015-1182-0

**Published:** 2015-06-09

**Authors:** Claudius U Meyer, Julia Birkholz, Nadine Weins, Aysefa Doganci, Stephan Gehring, Fred Zepp, Markus Knuf

**Affiliations:** Pediatric Immunology, Children’s Hospital, University Medical Center of the Johannes Gutenberg University Mainz, Obere Zahlbacher Str. 63, 55131 Mainz, Germany; Children’s Hospital of the Dr. Horst-Schmidt-Kliniken, Wiesbaden, Germany

**Keywords:** Interleukin-27, Newborn IL-12 deficiency, Dendritic cells, Childhood development, Viral infectious diseases susceptibility

## Abstract

**Background:**

Interleukin-27 (IL-27) has been described to be highly expressed during the very first days after birth, but secretion of IL-27 by dendritic cells during the course of childhood has not been described.

**Findings:**

In our present study we enrolled children (n = 55) in the range from 1 day of to 18 years of age and asked for a small whole blood sample. The capacity of dendritic cells to produce IL-27 during childhood was measured after whole blood culture with or without inflammatory stimuli. Results support recent findings of high IL-27 levels after birth and lowest levels in adults. Interestingly, we detected an interim peak production level at early adolescence.

**Conclusion:**

These data hint to prominent roles of IL-27 at the very start of post-natal life. Furthermore, a link has been given to so far not described immunological events during puberty.

**Electronic supplementary material:**

The online version of this article (doi:10.1186/s13104-015-1182-0) contains supplementary material, which is available to authorized users.

## Findings

Telling basic research immunologists, that a human newborn has difficulties to produce interleukin-12 (IL-12) leaves them with quite some astonishment. The generally accepted model is, that IL-12 is a pivotal and necessary factor to induce a sufficient and specific Th1-polarized cellular immune response against viral pathogens. Recent pediatric studies indicate that the capacity of dendritic cells, the main producers of IL-12 in adults, only reaches adult-like levels until close to the beginning of adolescence [1]. During the first months of life the substantial lack of IL-12 expression results in the well-known Th2-biased neonatal T cell immune response.

Another family member of IL-12-like cytokines, IL-27, has been recognized not only as an inhibiting factor to various T helper cell subsets, but also as a cytokine with a pronounced capacity to induce Th1 polarization, thus having the potential to physiologically support or be in place of IL-12, at least in part. Recently, we identified exceptionally high levels of IL-27 expression of dendritic cells derived from neonates [2]. To further characterize IL-27 production during childhood we collected whole blood samples from children aged between 0 and 18 years and investigated the capacity of dendritic cells to produce IL-27.

At the University Medical Center Mainz and at the Dr. Horst-Schmidt-Kliniken Wiesbaden for this observational study we enrolled 55 healthy subjects aged 0–18 years after. Additionally, samples from five adult volunteers were collected as a control group. The study has been approved by the local ethical board of the Dr. Horst-Schmidt-Kliniken Wiesbaden. We received informed consent from the parents of all subjects and additionally from those subjects older than 12 years of age. The adult participants gave informed consent for a blood withdrawal. The heparinized whole blood samples were collected, and processed within 2 h after collection. Briefly, whole blood samples were diluted with culture medium. Diluted cell suspensions were cultivated for 6 h at 37°C and 5% CO_2_ in the presence or absence of the stimulators IFNγ (50 µg/ml; Strathmann Biotec), LPS (10 µg/ml; InvivoGen), IFNγ + LPS, PolyIC (25 µg/ml; InvivoGen), ssRNA (5 µg/ml; InvivoGen) or SEB (10 µg/ml; Sigma Aldrich). To detect the capacity to express IL-27, dendritic cells were identified using flow cytometry (LSR II, BD Biosciences) by antibody labeling of the cell surface markers CD14, HLADR, CD11c and CD123 (all BD Biosciences) combined with an intracellular staining procedure for detection of IL-27-positive dendritic cells (Additional file 1). Frequencies of myeloid Dendritic Cells (mDCs; CD14^−^HLADR^+^CD11c^+^) and plasmacytoid Dendritic Cells (pDCs; CD14^−^HLADR^+^CD123^+^) were determined (Additional file 2: Figure S2). Percentage of IL-27-positive mDCs and pDCs were assessed using intracellular staining with anti-IL-27 antibody (R&D Systems; anti-EBI3). Furthermore we analyzed the IL-27 plasma concentration in a subset of the study group using ELISA (Additional file 1).

**Figure 1:**
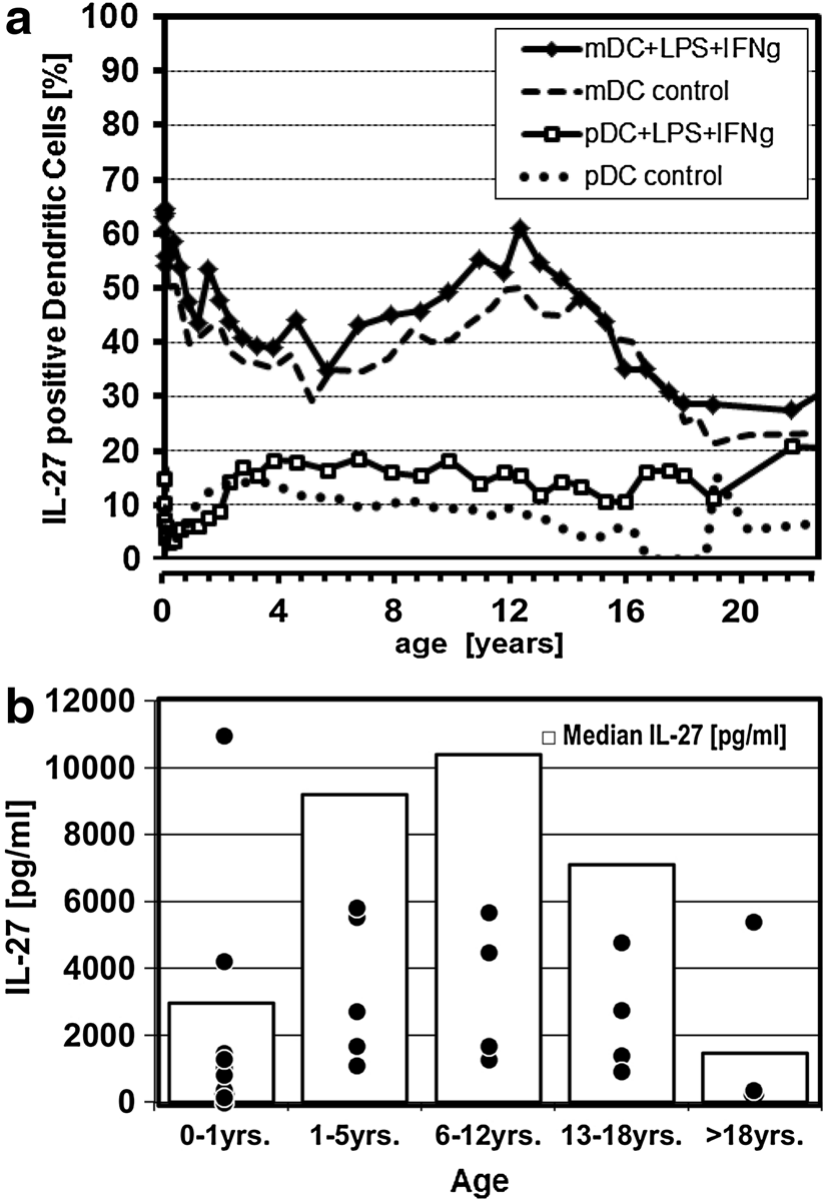
**a** Age-related frequencies of mDCs and pDCs producing IL-27. Age-based *simple moving mean* was applied as a trend indicator (see Additional file 4: Figure S4) to identify age-related differences in the capacity to produce IL-27 during childhood. Individual percentage of IL-27-positive mDCs were based on the CD14^−^HLADR^+^CD123^−^CD11c^+^ population for mDCs and CD14^−^HLADR^+^CD123^+^CD11c^−^ for pDCs (n = 41). **b** Age-related concentrations of IL-27 in plasma of peripheral blood. From a sub-set of subjects plasma was collected to test the concentration of IL-27. Levels of IL-27 in children less than 1 year of age were slightly higher as in adults, but considerably lower than IL-27 concentrations in the age groups between >1 and <18 years of age, with a maximum at about 6–12 years of age. (n > 5; except: 0–1 years, n = 12).

Whole blood collection from 41 subjects resulted in eligible sample volumes of at least 1 ml heparinized whole blood. Drop outs were related to technical difficulties to collect sufficient whole blood volume from neonates and very young infants. The frequency of both, mDCs and pDCs within the leukocyte population were stable during childhood, except for a slight decrease of less than three percent during the very first months after birth (Additional file 3: Figure S3). In contrast, high numbers of IL-27-positive mDCs were detected immediately after birth. In the following weeks and months these IL-27-positive mDC frequencies considerably decreased. Interestingly, during childhood and in contrast to an anticipated, rather linear decline over time, a second maximum was reached with a peak around the age range of 10–12 years. Our observation in adolescents and adults revealed, that IL-27 production levels were at lowest levels within the investigated cohort. This developmental kinetic was clearly detectable in unstimulated mDCs. Any of the used stimulators only slightly—if at all—modulated the underlying frequency of IL-27-positive mDCs with IFNγ + LPS being the most potent positive modulator (Figure 1a). pDCs presented with very low levels of IL-27 expression, specifically with no biphasic characteristics and no discernible difference regarding neonatal versus adult IL-27 levels (Figure 1a). IL-27 serum levels reached maximum levels in early adolescence in accordance with bisphasic IL-27-positive mDC frequencies (Figure 1b).

Comprehensive data have been accumulated on the limited expression of IL-12 in neonates [1, 3]. A contributing molecular mechanism of the latter phenomenon involves the histone component MUC2, which inhibits the accessibility of the *il12p35* gene [4]. Consequently, there is an obvious need for a compensating functional equivalent. Recent reports suggested several ways how IL-27 might be involved in STAT1-mediated Th1-like, e.g. antiviral immune responses [5]. IL-27 has been shown to induce the expression of the transcription factor T-bet, a gatekeeper factor for the transactivation of IFNγ and the IL-12ß2 receptor [6, 7]. This might foster a low level Th1 activity even in the presence of the minuscule levels of IL-12 seen in neonates. Moreover IL-27 plays a role in the generation of cytotoxic T cells, thus stimulating the expression of granzyme B in CD8^+^ T cells leading to augmentation of type 1 cell-mediated immunity. In addition, IL-27 is involved in the induction of regulatory T cells, like Tr1 and Tregs [8], and promotes the secretion of the Th2-cytokine IL-10 [9], all arguing for a role of IL-27 in ameliorating inflammatory processes.

Our finding of high IL-27 levels in early life might reflect the particular challenge of neonates adapting to the rapidly changing microbial community that starts developing immediately after birth. In a mutual interaction host immunity and the micro-flora starts to develop. The high levels of IL-27 in neonates coincided with the time period of maximum susceptibility for infectious diseases in childhood underpinning a critical role IL-27 may play. The presence of IL-27 correlates with the integrity of the mucosal lining and the resistance to translocation of microbes from the gut to the circulation [10].

A time frame in childhood with a second peak of elevated IL-27 expression was unanticipated. Interestingly, this developmental trait was not related to plasmacytoid cells, but otherwise it was also detectable when testing for IL-27 protein concentrations in peripheral blood plasma. To our knowledge, this observation has not been reported so far and in the light of the current literature focusing on the immune system of the developing child. Further investigations are prompted, linking this observation to other developmental patterns or disease dispositions in this age range.

In conclusion, our data on IL-27 indicate that during childhood development IL-27 does not represent a simple opposing trend to the IL-12 kinetics, but points to a more complex, yet in many details unknown regulative context.

## Additional files

Additional file 1:
**Text S1.** Detection of IL-27-positive dendritic cells and IL-27 in plasma. Additional file 2:
**Figure S2.** Detection of mDCs & pDCs in whole blood culture. Additional file 3:
**Figure S3.** Age-related cell frequency. Additional file 4:
**Figure S4.** Age related frequencies of IL-27-positive mDCs (top row) and pDCs (lower row).

## Abbreviations

DC: dendritic cells
mDC: myeloid dendritic cells
pDC: plasmacytoid dendritic cells
IL-27: interleukin-27
