# Supplementary material for: Dendritic cells change IL-27 production pattern during childhood
Source: BMC Res Notes. 2015 Jun 9;8:232. doi: 10.1186/s13104-015-1182-0 (PMC4467631; doi:10.1186/s13104-015-1182-0)
Supplement: Additional file 1: — Text S1. Detection of IL-27-positive dendritic cells and IL-27 in plasma. [file 13104_2015_1182_MOESM1_ESM.doc]

MS 3242122281298940

**Supplement S1:**

**Detection of IL-27 in Dendritic Cells**

Heparinized whole blood samples (16IU/ml Li-heparin) were taken from neonates, infants and children during clinical purpose venipuncture, and from adults during intentional venipuncture. Transportation of the whole blood samples to the lab was done at room temperature. Within 2 hours after blood was drawn, processing of blood was started in our lab. For whole blood cell culture 200µl blood were used per culture condition. This blood volume was diluted 1:1 with complete culture medium (RPMI 1640, supplemented with 10% heat-inactivated, thoroughly-tested fetal calf serum, 2 mM L-glutamine, 1 mM sodium-pyruvate, 1 x MEM (minimal essential medium)-amino acids, 100 IU penicillin and 100 µg/ml streptomycin and 50 µM b-mercaptoethanol) In case of medium control blood was diluted with complete culture medium without further additives. For culture conditions with stimuli whole blood was diluted with complete culture medium with one of the following additives: stimulators IFNy (50 µg/ml; Strathmann Biotec), LPS (10 µg/ml; InvivoGen), IFNy+LPS, PolyIC (25 µg/ml; InvivoGen), ssRNA (5 µg/ml; InvivoGen) or SEB (10 µg/ml; Sigma Aldrich). Additive concentrations had been tested in detail and already used for other projects as published for exp. in Krumbiegel et al. 2005, 2007 or 2008, or Birkholz 2014. With or without stimulator final dilution with culture medium was always 1:1 with the whole blood. Diluted cell suspensions were cultivated for 6 h at 37°C and 5 % CO_2_.

During the whole culture period Brefeldin A (10µg/ml, final concentration; f.c.) was present, as our aim was to collect all of the signals potentially stimulated by the stimulators. At the end of the 6 h culture period EDTA (f.c. 2mM) was added for 10 min before diluting the with FACS Lyse solution (BD Biosciences). After centrifugation the resulting cell sediment was washed with PBS, resuspended in PBS with Polyglobin (Bayer) and incubated for 10 min. Fluorochrome-labeled antibodies to surface proteins (anti-CD14, anti-HLA-DR, anti-CD123, and anti-CD11c) were added. After 20min at room temperature in the dark cells were washed with PBS and resulting cell sediment was resuspended BD Cytofix/Cytoperm (BD Biosciences) for 20min. Sedimented cells were resuspended in BD PWP with FITC-labelled anti-IL-27 antibody (R&D Systems) and incubated for 20min at room temperature in the dark. After stopping the intracellular staining by addition of a large volume of BD PWP, centrifugation and resuspension in PBS/FCS stained cell were analysed using a flowcytometer (LSR II, BD Biosciences; BD FACSDiva 6) thereby detecting 100.000 cells for each sample. Frequencies of myeloid Dendritic Cells (mDCs; CD14-HLADR+CD11c+) and plasmacytoid Dendritic Cells (pDCs; CD14^-^HLADR^+^CD123^+^) were determined using a gating strategy outlined in **S2**. Percentage of IL-27-positive mDCs related to total mDCs, as well as IL-27-positive pDCs related to total pDCs were collected.

**Detection of IL-27 in plasma**

IL-27 was detected for a subset of study participants using a commercial cytokine ELISA. IL-27 ELISA was used according to the procedures suggested in the manufacturer’s (R&D Systems).
