# Supplementary material for: Dendritic cells change IL-27 production pattern during childhood
Source: BMC Res Notes. 2015 Jun 9;8:232. doi: 10.1186/s13104-015-1182-0 (PMC4467631; doi:10.1186/s13104-015-1182-0)
Supplement: Additional file 2: — Figure S2. Detection of mDCs & pDCs in whole blood culture. [file 13104_2015_1182_MOESM2_ESM.pdf]

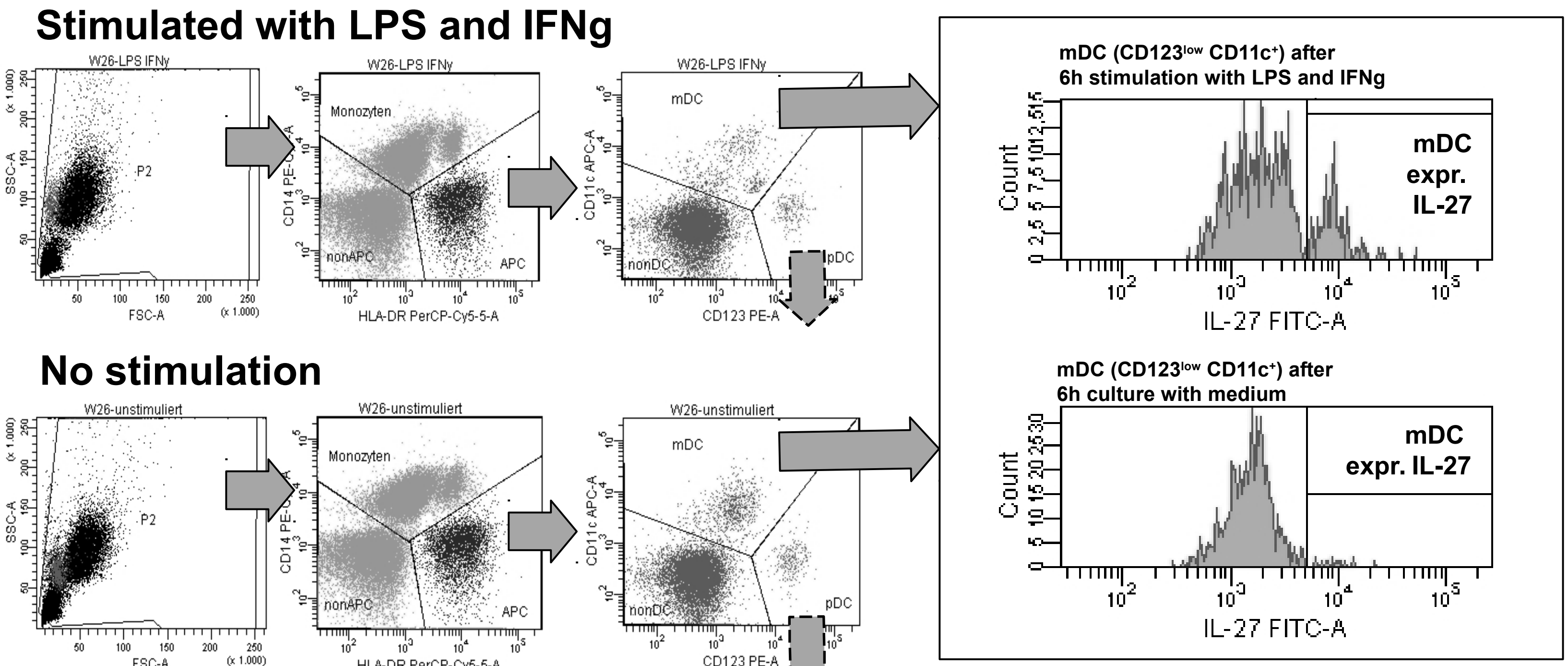

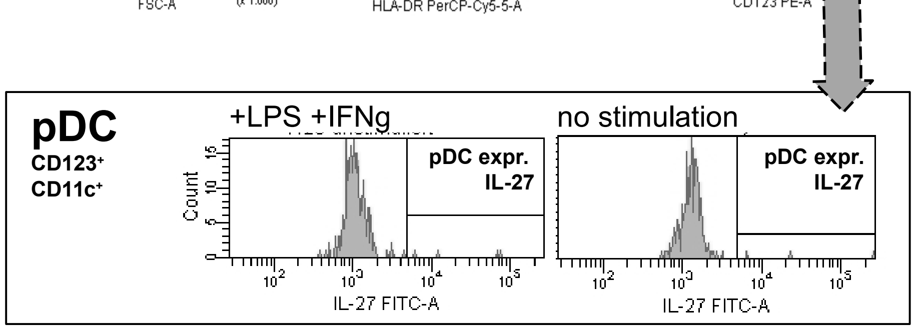

S2: Detection of mDCs & pDCs in whole blood culture.

Gating strategy to detect IL-27-positive mDCs & pDCs resulted in a histogram with a cut-off following visual plausibility. A typical result from a subject aged 11 years with a marked IL-27 response is depicted.
