# Supplementary material for: Dendritic cells change IL-27 production pattern during childhood
Source: BMC Res Notes. 2015 Jun 9;8:232. doi: 10.1186/s13104-015-1182-0 (PMC4467631; doi:10.1186/s13104-015-1182-0)
Supplement: Additional file 3: — Figure S3. Age-related cell frequency. [file 13104_2015_1182_MOESM3_ESM.pdf]

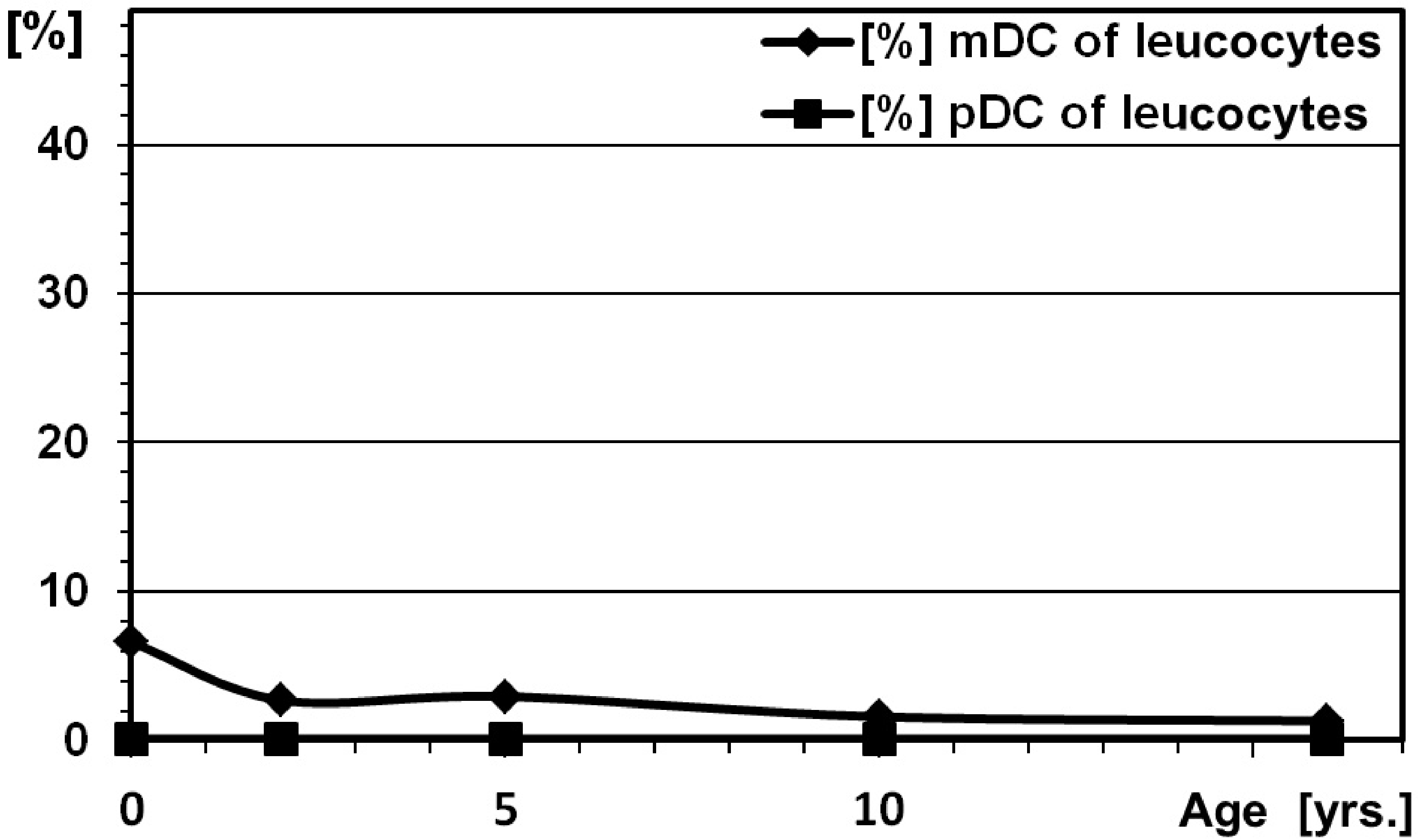

S3: Age-related cell frequency.

Frequencies of myeloid Dendritic Cells (mDC; $CD14^{-}HLADR^{+}CD11c^{+}$) and plasmacytiod Dendritic Cells (pDC; pDCs; $CD14^{-}HLADR^{+}CD123^{+}$) in age groups of study subjects indicated a slightly higher presence of mDCs early in life, but a steady level during childhood.
