# Supplementary material for: Dendritic cells change IL-27 production pattern during childhood
Source: BMC Res Notes. 2015 Jun 9;8:232. doi: 10.1186/s13104-015-1182-0 (PMC4467631; doi:10.1186/s13104-015-1182-0)
Supplement: Additional file 4: — Figure S4. Age related frequencies of IL-27-positive mDCs (top row) and pDCs (lower row). [file 13104_2015_1182_MOESM4_ESM.pdf]

mDC - medium control

a.) raw data IL-27-pos. cells [% of APCs]

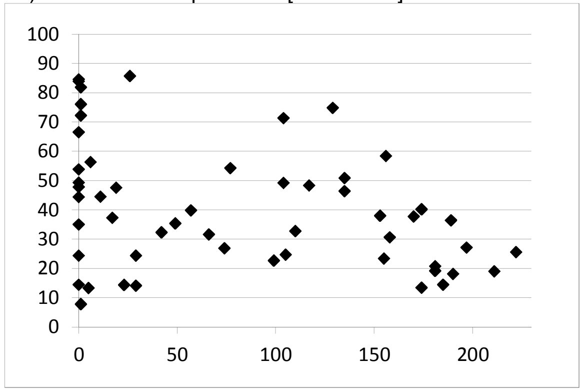

b.) Time-based *simple moving mean [% APCs-based]*

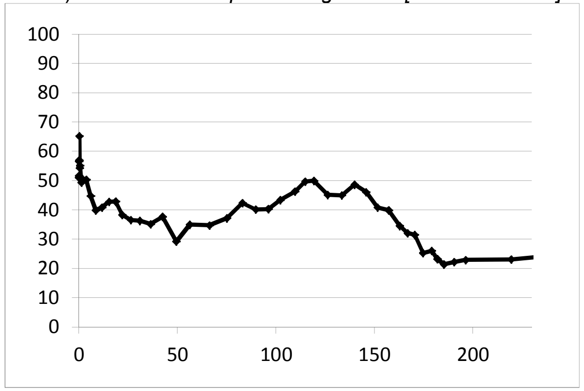

combination of a.) and b.)

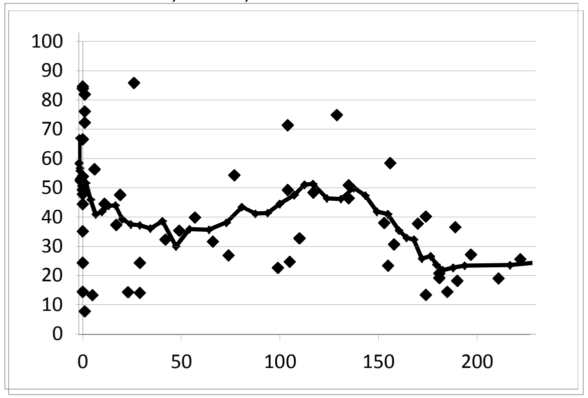

pDC - medium control

c.) raw data IL-27-pos. cells [% of APCs]

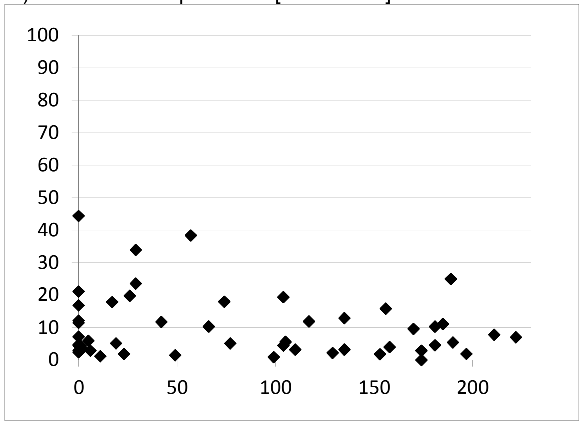

d.) Time-based *simple moving mean [% APCs-based]*

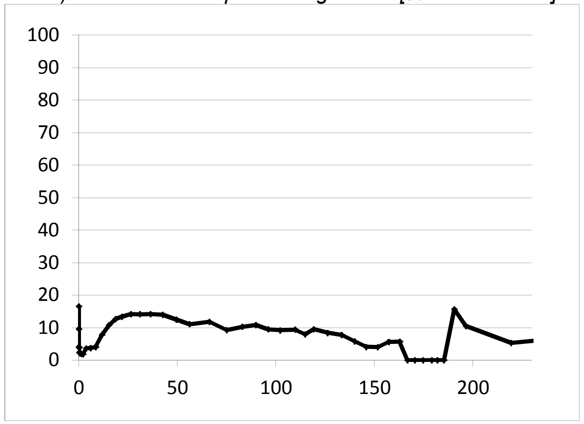

combination of c.) and d.)

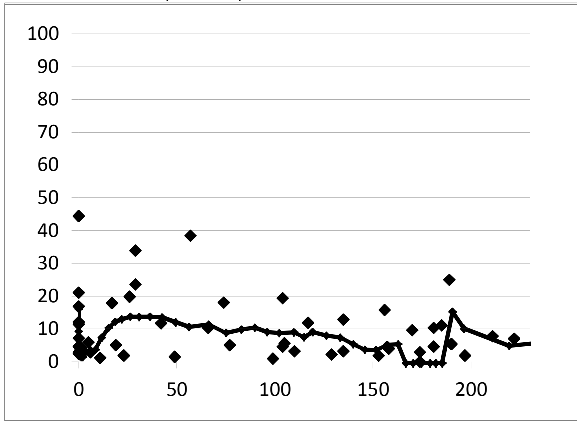

S4: Age-related frequencies of IL-27-positive mDCs (top row) and pDCs (lower row).
a.) and c.) percentages for each subject detected by flowcytometry, b.) and d.) calculation of ***time-based simple moving mean based on a calculation base of 6 sequential values*** has been applied to the percentage data to discern age-related **trends**. Visualization of combined view right panel each row.
